# Long‐term forecasting of blood donations using time series of donation activity: Findings from seven blood establishments

**DOI:** 10.1111/vox.70229

**Published:** 2026-03-09

**Authors:** Timo Asikainen, Tinus Brits, Ronél Swanevelder, Jose Antonio García Erce, Lucile Malard, Iris Garcia‐Martínez, Marijke Welvaert, Surendra Karki, Tea Lallukka, Mart Pothast, Mikko Arvas

**Affiliations:** ^1^ Finnish Red Cross Blood Service (FRCBS) Helsinki Finland; ^2^ Faculty of Medicine University of Helsinki Helsinki Finland; ^3^ South African National Blood Services Johannesburg South Africa; ^4^ Banco de Sangre y Tejidos de Navarra Navarre Spain; ^5^ Établissement français du sang La Plaine Saint‐Denis France; ^6^ Blood and Tissue Bank (BST) Catalonia Spain; ^7^ Vall d'Hebron Research Institute (VHIR), Universitat Autònoma de Barcelona (UAB) Catalonia Spain; ^8^ Australian Red Cross Lifeblood Sydney Australia; ^9^ Donor Health, Department of Research Sanquin Blood Supply Foundation Amsterdam the Netherlands

**Keywords:** blood donation, blood donation management, donor studies, long‐term forecasting

## Abstract

**Background and Objectives:**

To meet the long‐term demand for blood products while preserving donor health in the long run, blood establishments must recruit a sufficient number of new donors annually. To determine what will suffice, it is essential to be able to forecast future blood donation volumes as a function of new donors using estimates based on historical donation activity.

**Materials and Methods:**

Donor (*n* = 11,629,873) and donation data (*n* = 64,510,294) extracted from operational information systems of seven blood establishments were transformed into anonymous donation activity data (grouped by blood establishment, sex and blood group) in the form of time series of mean number of donations per donor, indexed by years since first donation. Linear models were fitted to the time series using ordinary least squares.

**Results:**

Out of the various estimated models, the best fit (mean *R* ^2^ over blood establishments 99.95%) for past mean donation activity was achieved when regressing the logarithm of cumulative donation activity on the logarithm of years since first donation and an indicator variable for the year of first donation. In addition to the predictions, comparison of the estimated parameters revealed that there are significant differences between blood establishments, translating to differences in the expected number of donations accumulated over the lifetime of a donor.

**Conclusion:**

Average donation activity can be modelled using a few variables and simple models, with high precision. The estimates can be used to create forecasts of future donation volumes, which in turn can be useful in long‐term blood donation management.

**Highlights**

- Average donation activity can be accurately modelled using a few variables and simple models, with high precision.
- Combined with the estimated number of future donors entering the donor pool, estimates of donation activity can be turned into forecasts of donation volumes.
- Forecast donation volumes are instrumental in long‐term blood donation management.

## INTRODUCTION

Blood establishments must match demand for blood products with adequate supply both in the short and long run. In the long run, the size of the donor pool and consequently the maximum donation capacity can be adjusted by changing how actively new donors are recruited. In the short run, a large donor pool enables reacting to sudden increases in demand. Additionally, a large donor pool is also beneficial for avoiding excessive donation pressure on specific groups of donors, such as O− donors. However, acquiring an overly large donor pool would require excessive resources and may be suboptimal from the management perspective. Hence, methods for finding the optimal size and influx to the donor pool are needed.

Previously, annual donor retention rates, donor recruitment rates and mean numbers of donations per donor and year have been used to estimate future blood donations in the Netherlands [1]. These supply estimates were further compared with two demand estimates based on population statistics and clinical blood use and were found to match the forecast demand. Similarly, in Japan, sex and age have been used as predictive factors in forecasting long‐term donations based on a Markov model, and the results were compared with population forecast–based estimates of demand for blood products [2].

Further, time series analysis has been applied to forecast blood use in England [3] and Finland [4]. In a similar vein, the prevalence of blood donation eligibility has been analysed in Australia: the eligible population forms an upper limit for how many donors can be recruited [5].

In this study, we develop a novel method for forecasting long‐term blood donation volumes using time‐series modelling of historical donation activity. Unlike the previous work on forecasting future donations, our work considers the entire known donation history of donors and can account for changes in the composition of the donor pool. In addition, the current study uses data from seven blood establishments instead of one. Combined with demand forecasts, blood establishments can employ our method to adjust their recruitment targets to meet the expected demand. Furthermore, we compare the estimates for average donation activity between seven blood establishments and discuss possible underlying factors and implications for donor management.

## MATERIALS AND METHODS

### Data

Seven national and regional blood establishments participated in the study:

1. Australian Red Cross Lifeblood (Australia)
2. Blood and Tissue Bank (BST, Catalonia, Spain)
3. Finnish Red Cross Blood Service (FRCBS, Finland)
4. Établissement Français du Sang (EFS, France)
5. Banco de Sangre y Tejidos de Navarra (Navarre, Spain)
6. Sanquin Blood Supply Foundation (the Netherlands)
7. South African National Blood Establishment (South Africa)

To use data from all donors and to enable as many blood establishments as possible to participate, the set of variables used in the study was restricted to the essential minimum. From donors, the following basic data were collected:

1. Date of birth
2. Sex
3. Blood group (A/B/O and Rh groups)

Similarly, for donations the following variables were used:

1. donorID
2. Date of donation
3. Type of place of donation: mobile or office

It was assumed that each donation is linked to a donor using a single identifier consistently, that is, donorID above. Only successful full‐blood donations were counted, except in South Africa where also cases in which less or more than the standard donation volume was drawn were counted. Summary data is presented in Table 1.

**TABLE 1:** Summary statistics of donors and donations.

	Australia	Catalonia	Finland	France	Navarre	The Netherlands	South Africa
Years	2010–2024	2000–2024	2000–2023	2015–2023	2000–2023	2009–2023	1993–2024
Donors (1000)	1470.84	1234.535	689.421	4052.891	19.995	809.05	3353.141
O−	8.3%	8.0%	4.9%	7.8%	11.1%	10.5%	4.1%
Female	55.0%	52.3%	54.8%	56.2%	53.4%	59.7%	51.1%
Age <25	40.2%	32.8%	38.5%	47.0%	78.2%	34.0%	55.5%
25 ≤ age < 40	31.6%	33.3%	28.9%	34.3%	20.8%	26.8%	28.0%
Donations (1000)	5912.443	6301.009	5048.547	15752.705	198.689	7088.737	24208.164
O−	13.5%	9.6%	6.8%	9.3%	13.2%	14.1%	6.2%
Female	51.6%	47.1%	50.8%	52.4%	34.1%	46.6%	41.8%
Age <25	39.7%	29.2%	27.9%	41.3%	61.4%	28.9%	47.0%
25 ≤ age < 40	26.5%	35.4%	29.4%	35.8%	37.8%	21.5%	31.4%

### Data collection and preprocessing

The variables used in this study are consistently collected and recorded in the operational information systems used in daily operations of the participating blood establishments. The data are at times extracted from the operational systems of blood establishments. The extracted data are truncated in time at the end and at the start, that is, the datasets may not include the entire history of donations within the blood establishments.

To avoid transferring donor and donation data containing sensitive personal data, the data for this study were collected as follows:

1. FRCBS provided a data description for a superset of data kinds and variables used in the study. The description can be found in the code repository for the project: https://github.com/FRCBS/donor-recruitment-prediction/.
2. Similarly, FRCBS provided R scripts that could be (a) modified to the datasets of a blood establishment to match the schema used in this project and to run consistency checks on the data and, once verified, (b) used to run the data summarization and export functionality described next to produce anonymous data files.
3. The resulting data were sent to FRCBS, where further analyses were done on the anonymous, aggregated data from the participating blood establishments.

**FIGURE 1:**
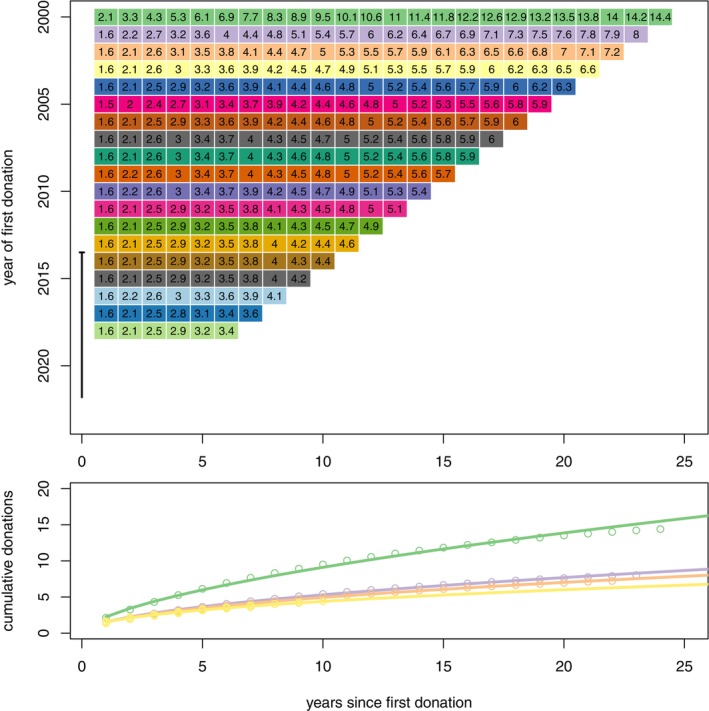
(Top) Example of a distribution matrix, and (bottom) models fitted (lines) to the rows of the distribution matrix (lines) and the original data (circles). The colours at the top and the bottom match each other. The predict future donations from the five final years (in yellow) and future years, a single model is fitted. In addition to these years themselves, additional years are included to achieve more accurate estimates for the parameters: These years are marked with the vertical bar between the matrix and axis.

The data per blood establishment were summarized as follows:

1. The donor and donation datasets were joined using *donorID* to form the (extended) donation dataset.
2. The date and year of first donation (*year0*) in the dataset were computed for each donor into dataset *donation0*.
3. The *donation0* dataset was joined with the extended donation dataset. For each donation, time since first donation (*diff*, in years) and the running number of the donations (*ord*) were computed by donor.
4. The donors were assigned to groups based on sex, age at first donation (under 25, 25–39, 40 or over), ABO and RhD blood groups (O−, other) and type of place of donation.
5. For each group and *year0*, the average number of cumulative donations per donor was computed for *x* = 1, 2, … years since first donation using the *diff* and *ord* variables computed above. Taken together, the averages form an upper‐triangular distribution matrix for the group with values of *year0* identifying rows and values of *x* columns. See Figure 1 for an illustration.
6. Size tables, that is, the number of new donors per year of first donation and for each group, were computed. Data pertaining to <30 people were removed.
7. The distribution matrices and size tables were exported as anonymous data and sent over for analysis.

For analysis, the distribution matrices were reorganized as follows. Each non‐empty cell in a distribution matrix constituted an observation. Moreover, the dependent variable was *donation activity*, that is, either the average cumulative number of donations during the first *x* years since first donation (*cdon*), or the number of donations during the *x*th year since first donation (*don*). Further, the main independent variable was *x*, the number of years (for *cdon*) or the sequential number of years since first donation (for *don*).

Figure 1 illustrates distribution matrices and how they correspond to time series for which models are fitted.

Initial inspection of the data revealed that the average number of donations per donor during the first year since first donation was on average higher than that of other years. This is, at least in part, explained by the fact that there is always a donation at the start of the first year. In contrast, a donor may not donate during a fixed period following the first donation, which attenuates the effect of the initial donation. Consequently, an indicator variable *x*_1_ (1 = first year, 0 = all other years) was added in the dataset to distinguish the first year from others.

In summary, the dataset used in the analysis consisted of the following variables:

1. group (defined by blood establishment, type of place, age and blood group)
2. year of first donation: *year0*
3. years since first donation: *x*
4. cumulative average number of donations: *cdon*
5. average number of donations: *don*
6. indicator of first year of donation: *x*_1_
7. group size

A description of the variables is also included in Table 2. As the number of groups is relatively large and the main focus lies in differences between blood establishment, sexes and blood groups, the data were further aggregated to three levels:

1. blood establishments
2. blood establishment × sex
3. blood establishment × blood group

**TABLE 2:** Estimated coefficients of determination (*R* ^2^) from different model specifications, data from the third year onwards.

Model	Australia	Catalonia	Finland	France	Navarre	The Netherlands	South Africa	Mean
*don* ~ *x.pwr*	0.94581	0.82126	0.87338	0.91669	0.71417	0.96316	0.89052	0.87500
log(*don*) ~ log(*x*)	0.95905	0.87096	0.91624	0.93120	0.69407	0.96024	0.92925	0.89443
log(*don*) ~ log(*x*) + *x*1	0.98241	0.95051	0.96338	0.99788	0.72125	0.97355	0.96007	0.93558
*don* ~ *x*.*pwr* + 0 + *year0*	0.97421	0.92110	0.94694	0.97048	0.91832	0.99118	0.95854	0.95440
*cdon* ~ *x*.*pwr*	0.99082	0.91029	0.98716	0.99110	0.93177	0.98325	0.91127	0.95795
*cdon* ~ *x*.*pwr* + *x*1	0.99184	0.91383	0.98765	0.99116	0.93178	0.98327	0.91131	0.95869
*don* ~ *x*.*pwr* + *x*1	0.99414	0.98540	0.99192	0.99761	0.78054	0.99006	0.97723	0.95956
log(*cdon*) ~ log(*x*)	0.98938	0.94226	0.99152	0.99017	0.91228	0.98845	0.93849	0.96465
log(*cdon*) ~ log(*x*) + *x*1	0.99055	0.94681	0.99256	0.99025	0.91303	0.98849	0.93866	0.96577
log(*don*) ~ log(*x*) + 0 + *year0*	0.98499	0.98699	0.98525	0.96367	0.91183	0.98917	0.99226	0.97345
log(*don*) ~ log(*x*) + 0 + *year0* + *x*1	0.99373	0.99776	0.99424	0.99933	0.91802	0.99399	0.99842	0.98507
*don* ~ *x.pwr* + 0 + *year0* + *x*1	0.99842	0.99721	0.99798	0.99958	0.93693	0.99849	0.99633	0.98928
*cdon* ~ *x*.*pwr* + 0 + *year0*	0.99984	0.99778	0.99964	0.99994	0.99689	0.99943	0.99881	0.99891
*cdon* ~ *x.pwr* + 0 + *year0* + *x*1	0.99988	0.99790	0.99968	0.99998	0.99692	0.99946	0.99881	0.99895
log(*cdon*) ~ log(*x*) + 0 + *year0*	0.99989	0.99930	0.99980	0.99991	0.99831	0.99979	0.99992	0.99956
log(*cdon*) ~ log(*x*) + 0 + *year0* + *x*1	0.99991	0.99947	0.99988	0.99998	0.99838	0.99982	0.99992	0.99962

*Note*: don, annual donation activity (donations per donor per year); cdon, cumulative donation activity; x, number of the year; x.pwr, number of the year (*x*) converted using the power function, the exponent estimated from data; x1, indicator variable for year being the first year of donations; *year0*, factor with years of first donation as level; log(·), the (natural) logarithm function; 0, no intercept term included in the model (by default an intercept is included).

When aggregating, the donation activity variables *cdon* and *don* were summarized as weighted averages, with group sizes as weights.

### Analysis

The purpose of the analysis was to model the observed donation activity and make predictions of future donation activity. The time series of donations stemming from each year of first donation (*year0*) were forecast separately and summed thereafter.

Due to data truncation, the first donation appearing in the data was in many cases actually not the donor's first donation. Consequently, the donation activity and the corresponding distribution matrices are not alike for all *year0*'s: these years include repeat donors, who are, on average, more active than first‐time donors. This phenomenon is especially prominent during the first years of data. Based on inspection of the data, the first 2 years were estimated separately.

Further, due to the way the data was constructed, the final year *x* (year since first donation) was dropped from each row (identified by *year0*) of the distribution matrix, as the number of cumulative donations did not include a full year of donation data by construction.

To achieve the best possible fit for the data, the following design questions were considered and the results compared. First, we considered different approaches to whether models should be fit for different *year0*'s separately or together, and to what extent. If it is assumed that donation activity has remained the same through time, estimating a single model should yield the most accurate estimates. However, estimating each *year0* separately can be expected to work better if there are substantial changes in donation activity.

The final years of the data must be estimated together to ensure a sufficient number of data points to estimate parameters. In addition, when estimating the final years, additional years termed *overlap* (*years*) can be included in the model for improved accuracy even though the overlap years would be predicted separately.

Second, we studied different functional forms for the regressions. Either the form

$$\text{cdon}|\mathrm{don}\sim a\cdot x^{b}+\text{auxiliary variables},$$

termed the *power form*, or

$$\log\left(\text{cdon}|\mathrm{don}\right)\sim \log\left(a\right)+b\;\log\left(x\right)+\text{auxiliary variables},$$

termed the *logarithmic form*, can be used. In both forms, *cdon|don* is either *cdon* or *don*, *a* (also termed *multiplier*) is a parameter increasing with the donation activity and *b* (also termed *exponent*) is a parameter pertaining to the steepness of the donation activity curve. The auxiliary variables may include a *year0* factor and the *x*_1_ indicator variable, as described above.

Third and finally, closer inspection of the data also revealed that the donation activity has fluctuated significantly for some blood establishments and calendar years, constituting upward spikes in the time series of actual donations. When estimating the time series of donation activity, this causes dual complications. First, as the spikes occur in multiple time series, they cannot be estimated using our approach so that the forecasts would match the actual spiked data. Second, the spikes will nevertheless distort the estimates, resulting in the donation activity outside the spike being overestimated.

To mitigate these effects, a method for filtering the largest deviations from the data was devised: when estimating a model, all the points where the proportion of the absolute error (value − fitted value) to the fitted value exceeded a given threshold *r* were removed from data and the model was re‐estimated with the modified data.

In the final analysis, the data were split in two alternative ways:

1. The first 2 years were estimated *separately*, and the succeeding years together.
2. The final 5 years of data were estimated *together* with up to 5 years of overlap, and the preceding years separately.

For both ways to split the data, models for each functional form and set of auxiliary variables were estimated. For the logarithmic form, the log‐transformations were made, parameters were estimated using ordinary least squares and predictions were retransformed using the exponent function. Further, the estimated models were used to predict activity profiles with 95% confidence intervals covering years 1–55 since first donation.

### Forecasting donations

The number of new donors per year was used in conjunction with the activity profiles to determine donation forecasts. For years with the actual number of new donors available, this number was used. For future years, the activity profiles and the number of new donors from the last year with data were used. In addition, a *no‐new‐donors* scenario was computed, with the assumption that no new donors enter the pool beyond the known new donors.

All analyses were run using the R statistical software [6] version 4.4.2.

## RESULTS

### Forecasted donations and related time series

**FIGURE 2:**
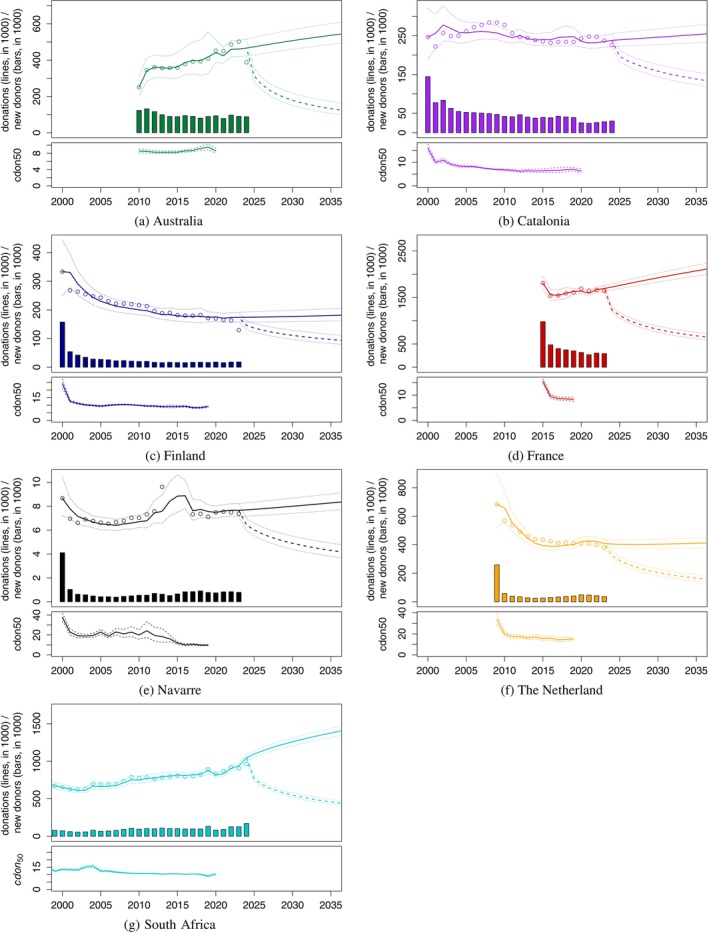
Summary of estimated models and forecast donations by country (a–g). In each figure, the top panel contains the actual donation amounts (circles), estimated donations assuming a constant number of annual new donors, and their confidence intervals (solid and dotted lines) and number of new donors (bars); an alternative scenario of forecast donations with no new donors entering the donor pool is shown with dashed lines with confidence intervals (dotted lines). Further, the bottom part contains statistics about donor activity: The *cdon*_50_ values and their confidence intervals are shown in solid and dashed lines, respectively.

Figure 2 contains details of the forecasts based on the log(*don*) ~ log(*x*) + *x*_1_. Overall, based on Figure 2, the fluctuations in the actual number of donations can be attributed to changes in donation activity: important peaks in these time series do not coincide with similar peaks in the number of new donors. Overall, the number of new donors is relatively stable over time, except for the larger numbers during the first couple of years of data for each country: this is due to existing donors appearing in the data for the first time and counted as new donors.

The differences in parameters discussed above are also reflected in Figure 2. In addition to the differences in *cdon*_50_‐levels, blood establishments with low *cdon*_50_‐values also exhibit sharp drops in the no‐new‐donors scenario: in essence, high activity of repeat donors helps maintain a steady supply of donations.

### Estimated parameters of donation activity

**FIGURE 3:**
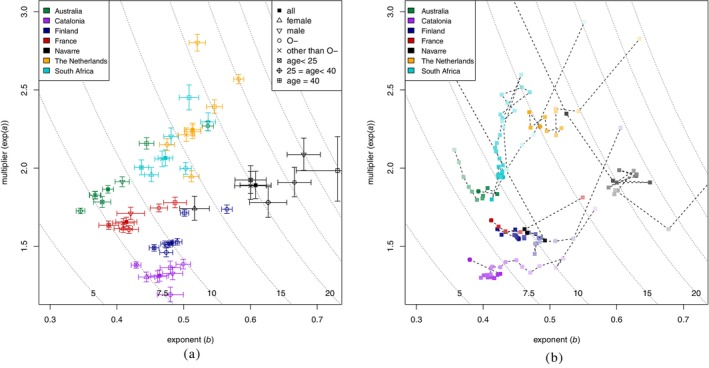
Estimated parameters for different blood establishments from model log(*cdon*) ~ *a* + *b*log(*x*), where *b* and exp(*a*) are referred to as the exponent and multiplier and plotted on the horizontal and vertical axes, respectively; see legends in figure for the symbols used. In addition, the figure includes contours (dotted curves) along which the expected number of cumulative donations in 50 years (*cdon*_50_) is constant. Respective constant values for contours are given above the horizontal axis. (a) Estimates from model where all the years taken together (third year onwards). Significant differences between blood establishments and between groups within blood establishments can be observed. The dotted curves are contours of the estimated cumulative donations in 50 years. (b) Parameters for blood establishments estimated separately for each year in data. Dark colours represent more recent years.

The parameter estimates for the log(*cdon*) ~ log(*x*) models estimated for each blood establishment are illustrated in Figure 3a. The figures also include the estimated parameters for the subpopulations formed based on the dimensions sex and blood group.

The figure highlights that there are significant differences between different blood establishments and between groups within the same blood establishment. In general, males donate more actively than females, and the O− donors are particularly active. Pertaining to age, donors with first donation at age of 40 or older are the most active group. Also, for some blood establishments, for example, Catalonia and Navarre, there is significant variance from year (of first donation) to year (Figure 3b), whereas others, for example, Australia and Finland, exhibit more stable trajectories.

### A spreadsheet‐based prediction tool

To support developing and running different scenarios by different stakeholders, we developed a spreadsheet tool, available at the project repository https://github.com/FRCBS/donor-recruitment-prediction.

### Model performance

**FIGURE 4:**
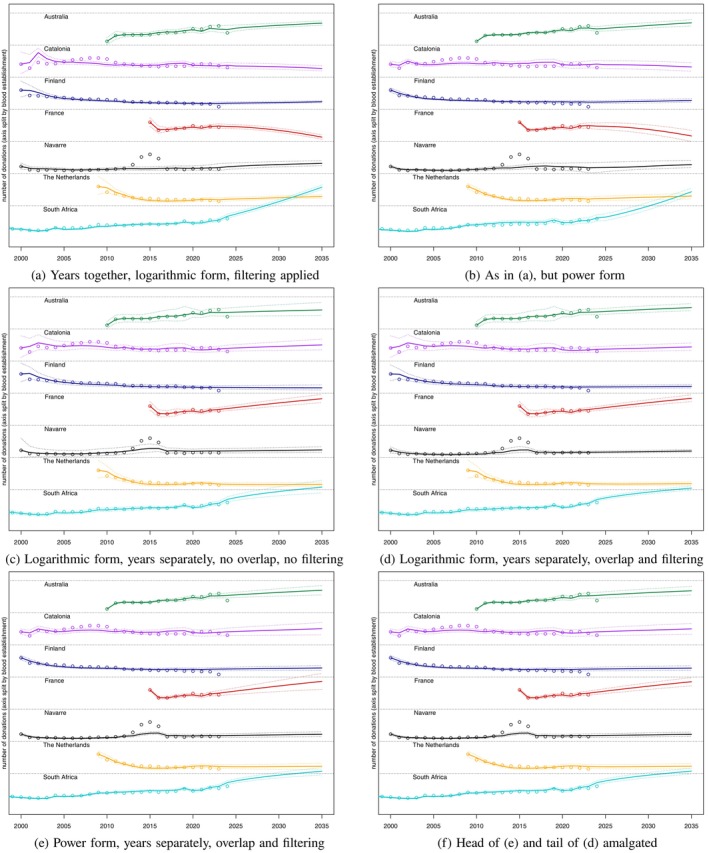
Forecasts with different functional forms and model structures. The illustrations for each blood establishment have been scaled to roughly fit the designated height while keeping the positions of the actual donation symbols fixed between the panels. (a) All years are estimated together as a single model using the logarithmic form, filtering applied. The forecast errors exhibit some serial correlation, but, overall, the forecasts are the most accurate, resulting in the tightest confidence intervals of all the alternatives discussed in this figure. (b) Similar, but with the power form. The confidence intervals for forecasts are significantly wider for this specification, although the predictions for the past values are more accurate. (c) All years but the last five estimated separately, no filtering applied and no overlap for the tail. The lack of filtering causes the spikes, for example, around year 2015 for Navarre, to distort the forecast both before and after the spike, leading to overly large estimates outside the spike, and underestimation during the spike. (d) All years but the last five estimated separately, both filtering and overlap at the tail applied. The additional effect of overlap affects the confidence intervals at the right tails, especially prominently for Australia and the Netherlands. (e) The power functional form yields better forecasts for the years estimated individually but is inferior to logarithmic form at the tails; filtering and overlap applied. (f) The tail from panel (d) combined with the head from panel (e). Note that the tail also includes predictions from the head.

On comparing the performance of different models, Figure 4 contains the forecast donations with confidence intervals for a number of model structures and functional forms. Further, Table 2 contains the coefficients of determination (*R* ^2^) from different functional forms described above. The figure suggests that adjustments, filtering in particular, and to a lesser extent adding overlapping data when estimating parameters for the tail years, affect the quality of the forecasts more significantly than the choice between using either the logarithmic or the power form. This is supported by Table 2, which shows that the differences in *R* ^2^ values between the logarithmic and power forms are generally small given the same auxiliary variables. Additionally, Figure 2 shows that the power form tends to perform better, that is, resulting in narrower confidence intervals, for the initial years since first donation, but the logarithmic form performs better for the forecasts of the future. For this reason, the logarithmic form from Figure 4d, that is, all years but the last five estimated separately, with both filtering and overlap at the tail applied, is used in further analyses below.

## DISCUSSION

The data used in this study are rather simple, consisting only of the very basic donor and donation records of the participating blood establishments. In particular, no information about the relevant blood donation management processes or changes in organization of the blood establishments was included: requiring such information would have significantly reduced both the blood establishments and years that have comparable data. However, despite the rather minimal knowledge base, we were able to realize high‐quality forecasts. The results show that donation activity remains relatively stable over time despite major events affecting many aspects of societies, such as the COVID‐19 pandemic.

Several modelling choices were made during the analysis. First, we opted not to explicitly include the effect of mandatory attrition due to age. Experimenting with such models resulted in inferior model performance. This can be explained by the fact that the observed time series already include the effect of attrition due to age. Including it explicitly in the model would, therefore, result in overestimating its effect. Second, the time series were censored by filtering out extreme values in the time series. This is justified by the fact that the purpose was to determine long‐term forecasts concerning the normal, steady state of operations, whereas the spikes that were filtered out were caused by exceptional circumstances that are not expected to be repeated in the future.

The differences observed in the parameter estimates between blood establishments, as illustrated in Figure 3, have some well‐known explanations. Females are more prone to adverse events, which may preclude them from becoming a return donor [7]. As the universal donor blood group, O− blood is indispensable in emergency transfusions, and therefore blood establishments must maintain adequate capacity to supply it when needed. Consequently, group‐O− donors are encouraged to donate frequently.

Interestingly, there are major differences in the donation activity between blood establishments. In particular, the donation activity in the Netherlands, Navarre and South Africa consistently exceeded the activity for other blood establishments. The data as such do not yield satisfactory answers why these differences occur. However, as none of the corresponding societies is known to be significantly different from the other participants, the most probable explanation for the differences lies in differences in blood donation management—in particular retention management—as differences in management of iron status of donors are not likely to explain these results [8, 9]. During recent years, in the Netherlands and Navarre, the implementation of patient blood management programmes and an increase in apheresis donations have reduced the demand for red blood cells, and as a result, less supply and fewer donations are needed.

Predictions for future donation activity can be created based on the estimated donation activity, that is, the number of donations per donor/year. For the first predicted years, the estimated annual donations will stem mainly from existing donors whose behaviour has been used to estimate the models. However, when estimating further into the future, the balance will shift towards (1) donations from existing donors many years since their first donation, which implies larger margins of error, and (2) donations from donors where data is not available yet and the number of which is unknown and depends mostly on the number of new donors that are recruited, and ultimately on the demand for blood products and the resources that are available for the recruitment.

The quality of forecasts, defined in terms of the width of the confidence intervals, depends essentially on the quality of data. As the results in Figure 2 show, a long time series of data beyond the essential minimum of approximately 7 years is neither a requirement nor a guarantee for high‐quality predictions. Even after the removal of outlying data points through filtering, there were differences in the quality of predictions between blood establishments, due to differences in variance: these differences are not explained by the number of donations. Finally, the quality of predictions with adjustments for all participating blood services was found to be good or excellent.

To the best of our knowledge, no previous study has followed the same or even similar approach to modelling past donation activity using time series analysis. However, some previous work with similar methodology and/or subject exists. In studies similar to this, first or first few donations have been given special emphasis in predicting long‐term donations. First‐year donations have been shown to predict long‐term commitment [10]. Planned behaviour theory has been applied to predict first‐time donor retention in Australia [11] and Ireland [12]. Finally, impact of the first donation experience on developing a commitment to blood donation has been studied and found relevant [13].

A significant limitation of this study is that it only addresses long‐term predictions for supply of blood. However, blood establishments additionally need tools to predict short‐term fluctuations in blood supply and demand. Developing models and tools for such predictions is an important topic for future work.

In conclusion, a look into the historic donation data reveals that the trajectory of donation activity in years since the first donation is highly predictable and follows the same functional forms in all the participating blood establishments. This observation also enables the creation of trustworthy long‐term forecasts of donations. However, there are significant differences between the estimated parameters for different blood establishments, most likely due to different blood donation management procedures. The provided spreadsheet tool and parameter estimates allow blood establishments to create scenarios by changing the forecast number of new donors and donor activity used in creating the forecasts.

## CONFLICT OF INTEREST STATEMENT

The authors declare no conflicts of interest.

## Data Availability

The data that support the findings of this study are available on request from the corresponding author. The data are not publicly available due to privacy or ethical restrictions.

## References

[1] Borkent‐Raven BA , Janssen MP , Van Der Poel CL . Demographic changes and predicting blood supply and demand in the Netherlands. Transfusion. 2010;50:2455–2460. 20529000 10.1111/j.1537-2995.2010.02716.x

[2] Akita T , Tanaka J , Ohisa M , Sugiyama A , Nishida K , Inoue S , et al. Predicting future blood supply and demand in Japan with a Markov model: application to the sex‐and age‐specific probability of blood donation. Transfusion. 2016;56:2750–2759. 27595917 10.1111/trf.13780

[3] Nandi AK , Roberts DJ , Nandi AK . Improved long‐term time‐series predictions of total blood use data from England. Transfusion. 2020;60:2307–2318. 32691487 10.1111/trf.15966

[4] Turkulainen EV , Wemelsfelder ML , Janssen MP , Arvas M . A robust autonomous method for blood demand forecasting. Transfusion. 2022;62:1261–1268. 35383944 10.1111/trf.16870 PMC9325496

[5] Mowat Y , Hoad V , Haire B , Masser B , Kaldor J , Heywood A , et al. Prevalence of blood donation eligibility in Australia: a population survey. Transfusion. 2023;63:1519–1527. 37464879 10.1111/trf.17474 PMC10952191

[6] R Core Team . R: a language and environment for statistical computing. 2021. Available from: https://www.r‐project.org/. Last accessed 26 Feb 2026.

[7] Newman B . Iron depletion by whole‐blood donation harms menstruating females: the current whole‐blood‐collection paradigm needs to be changed. Transfusion. 2006;46:1667–1681. 17002622 10.1111/j.1537-2995.2006.00969.x

[8] van den Hurk K , Arvas M , Roberts DJ , Castrén J , Erikstrup C . Whole blood donor iron management across Europe: experiences and challenges in four blood establishments. Transfus Med Rev. 2024;38:150860. 39369584 10.1016/j.tmrv.2024.150860

[9] Vinkenoog M , Toivonen J , Brits T , de Clippel D , Compernolle V , Karki S , et al. An international comparison of haemoglobin deferral prediction models for blood banking. Vox Sang. 2023;118:430–439. 36924102 10.1111/vox.13426

[10] Schreiber GB , Sharma UK , Wright DJ , Glynn SA , Ownby HE , Tu Y , et al. First year donation patterns predict long‐term commitment for first‐time donors. Vox Sang. 2005;88:114–121. 15720609 10.1111/j.1423-0410.2005.00593.x

[11] Masser BM , Bednall TC , White KM , Terry D . Predicting the retention of first‐time donors using an extended theory of planned behavior. Transfusion. 2012;52:1303–1310. 22257135 10.1111/j.1537-2995.2011.03479.x

[12] McMahon R , Byrne M . Predicting donation among an Irish sample of donors and nondonors: extending the theory of planned behavior. Transfusion. 2008;48:321–331. 18028275 10.1111/j.1537-2995.2007.01526.x

[13] Callero PL , Piliavin JA . Developing a commitment to blood donation: the impact of one's first experience. J Appl Soc Psychol. 1983;13:1–16.

